# Surface Piezoelectricity and Pyroelectricity in Centrosymmetric Materials: A Case of α-Glycine

**DOI:** 10.3390/ma13204663

**Published:** 2020-10-19

**Authors:** Shiri Dishon, Andrei Ushakov, Alla Nuraeva, David Ehre, Meir Lahav, Vladimir Shur, Andrei Kholkin, Igor Lubomirsky

**Affiliations:** 1 Department of Materials and Interfaces, Weizmann Institute of Science, Herzl St 234, Rehovot 7610001, Israel; shiri.dishon@weizmann.ac.il (S.D.); david.ehre@weizmann.ac.il (D.E.); meir.lahav@weizmann.ac.il (M.L.); 2 School of Natural Sciences and Mathematics, Ural Federal University, Lenin Ave. 51, 620000 Ekaterinburg, Russia; andrey.ushakov@urfu.ru (A.U.); alla.nuraeva@urfu.ru (A.N.); vladimir.shur@urfu.ru (V.S.); 3 CICECO-Aveiro Institute of Materials, Department of Physics, University of Aveiro, 3810-193 Aveiro, Portugal

**Keywords:** surface pyroelectricity, surface piezoelectricity, α-glycine

## Abstract

Surface pyroelectricity and piezoelectricity induced by water incorporation during growth in α-glycine were investigated. Using the periodic temperature change technique, we have determined the thickness (~280 µm) of the near surface layer (NSL) and its pyroelectric coefficient (160 pC/(K × cm^2^) at 23 °C) independently. The thickness of NSL remains nearly constant till 60 °C and the pyroelectric effect vanishes abruptly by 70 °C. The piezoelectric effect, 0.1 pm/V at 23 °C measured with an interferometer, followed the same temperature dependence as the pyroelectric effect. Abrupt disappearance of both effects at 70 °C is irreversible and suggests that water incorporation to α-glycine forms a well defined near surface phase, which is different form α-glycine because it is polar but it too close to α-glycine to be distinguished by X-ray diffraction (XRD). The secondary pyroelectric effect was found to be <14% of the total, which is unexpectedly small for a material with a large thermal expansion coefficient. This implies that water incorporation infers minimal distortions in the host lattice. This finding suggests a path for the control of the piezoelectric and pyroelectric effects of the crystals using stereospecific incorporation of the guest molecules.

## 1. Introduction

The crystal surface symmetry and physical properties are very important in a wide variety of applications, for example: using a suitable substrate can control symmetry and epitaxial growth of thin films for various applications in microelectronics, microelectromechanical systems (MEMS), catalysis and more. It has been reported that some centrosymmetric crystals contain a near surface layer (NSL) with polar symmetry. These layers were detected by pyroelectric measurements [1,2,3] or suggested by theoretical calculations [4,5,6]. Piezoelectric properties of such layers are frequently detected by Piezoresponse Force Microscopy (PFM) that is, in particular, sensitive to surface piezoelectricity [7,8,9]. The most popular interpretation of the surface piezoelectricity was a polarization arising due to strain gradient at the surface, i.e., flexoelectric effect. It can be caused, for example, by the PFM tip, that may cause highly inhomogeneous stress/strain just below the contact [10]. However, it is very hard to find any reliable experimental report on surface piezoelectricity in dielectric centrosymmetric crystals, although every non-centrosymmetric layer should give a piezoelectric response. The main reason for the lack of surface piezoelectric measurements is that even an NSL with a large piezoelectric coefficient, *d_ii_*, will give a small displacement, u, even at high applied voltage as can be explained as follows. If the electric capacity and conductivity of the NSL are similar to those of the bulk crystal, the electric field inside the sample is constant and equal to U/$Th_{s}$, where U is the external applied voltage and $Th_{s}$ is the sample thickness including the thickness of the NSL. In this case, the piezoelectric displacement will be described by [11]:

(1) $$u=d_{22}\times U\times \frac{\delta_{s}}{Th_{s}}$$

where $\delta_{s}\;$is the NSL thickness and $d_{22}$ is the piezoelectric coefficient in the polar direction in polar monoclinic crystals (subscript “2” indicates that it is in b-direction of the crystal (010)). Since for most single crystals$\;\frac{\delta_{s}}{Th_{s}}\;$is in the order of 10^−5^, one should apply tens of thousands of volts to get a measurable displacement. Since, $\delta_{s}$ is usually unknown, the value that can be deduced from the experiment is:

(2) $$d_{22}\times \delta_{s}=\frac{uTh_{s}}{U}$$

One exception to the above claim is α-glycine (α-gly), which has a monoclinic centrosymmetic space group $P2_{1/n}$ with four molecules per unit cell (a=5.105 Å, b=11.97 Å, c=5.465 Å, α=γ = 90°, β = 111.7°). In contrast to inorganic crystals like SrTiO_3_ [12], where the expected NSL is < 2 nm thick, the thickness of the surface polar layer in α-gly, like in other amino acid crystals, may exceed 100 microns [1,2,3,13]. The NSL is so thick in α-gly because a reduction in symmetry arises due to a partial water incorporation during the crystal growth [2]. This makes α-gly a very suitable candidate for the experimental verification of the existence of the surface piezoelectricity in crystals. Measuring the surface piezoelectricity in α-gly may answer two important questions:

(a) What is the magnitude of the surface piezoelectric effect, which will allow discrimination between the primary and secondary contributions to pyroelectricity.
(b) Does the surface piezoelectric effect vanishes concurrently with the pyroelectric effect or persists for some time after the latter disappears with temperature? If yes, would it imply that the structure first loses polarity but remains for some time non-polar but non-centrosymmetric.

Here, we report the results of the direct measurements of the temperature dependence of the piezoelectric effect in single crystals of α-gly using a Michelson–Morley laser interferometer. 

## 2. Materials and Methods 

The single crystals of α-gly were grown by slow evaporation technique from aqueous solution using a 100 mL of deionized water (Merck Millipore, Burlington, MA, USA, 18.2 MΩ cm at 23 °C Type 1 water) and 30 gr of the glycine powder (Sigma-Aldrich, St. Louis, MO, USA, ≥99%) as described previously [1,2]. In order to achieve complete dissolution of glycine powder, the solution was heated up to 75–80 °C and stirred for 3 h. After the solution became completely transparent, it was filtered through cotton wool to the crystallization dishes (about 30–35 mL per one dish) and left for 12–17 h. The as-grown crystals were transparent and had a typical for α-gly natural habitus with a clearly expressed {010} planes, which was earlier reported to exhibit pyroelectricity [1,2]. The typical thickness of the crystal was 3–10 mm. 

The as-grown crystals were cut parallel to the top exposed {010} face into plates. Then, silver paste electrodes (VS Electronic Vertriebs, Aschaffenburg, Germany) were applied on both, top (as-grown) and bottom (sliced), {010}-faces. Four batches of crystals were grown and examined. For the as-grown surfaces, less than 30 min elapsed between the removal of crystals from the growth solution and commencing the measurements. As a control, some of the crystals were cleaved parallel to {010} faces at least 0.3 mm below the naturally grown surface.

The displacement (in response to the applied voltage) was measured with a single-beam Michelson–Morley interferometer [14,15,16] equipped with a SR830 lock-in amplifier (Stanford Research Systems Inc., Sunnyvale, CA, USA). A proportional-integral-derivative (PID) feedback system consisting of P-841.01 piezo actuator and E-709.SRG piezo controller (Physik Instrumente, Karlsruhe, Germany) was used for the working point stabilization against the slow changes of the optical path length. A heating stage made of titanium film as a heating element clamped between two copper plates of 30 × 30 × 1.5 mm^3^ size was utilized for the temperature-dependent measurements. The temperature stabilization system was based on Pt100 class B resistance temperature sensor (Heraues Nexensos, Kleinostheim, Germany), MB110 input-output programming module (Owen, Moscow, Russia), and ZUP60-3.5 programmable power supply (TDK-Lambda Corporation, Tokyo, Japan). The heating stage was mounted on a polytetrafluoroethylene base, thermally isolated from the rest of the system with polyurethane foam. The measurements were carried out under excitation signal with amplitude of 50–150 V and frequency of 6–8 kHz, which is high enough to eliminate interference from mechanical resonances and electromagnetic noise. Every data point was obtained by averaging at least three measurements of the displacement amplitude. 

The surface pyroelectric coefficient and the thickness of the NSL were measured using the periodic temperature change (PTC) as described in refs. [2,11]. 

## 3. Results and Discussions

### 3.1. Pyroelectric Measurements

**Figure 1:**
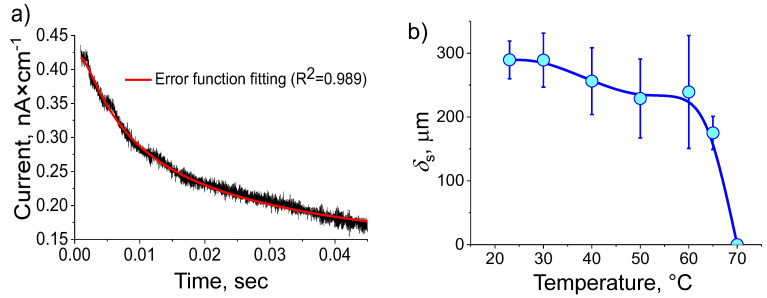
(**a**) Decay of the pyroelectric current with time *j(t*), at 35 °C; (**b**) Temperature dependence of the NSL thickness$\;\delta_{s}$.

**Figure 2:**
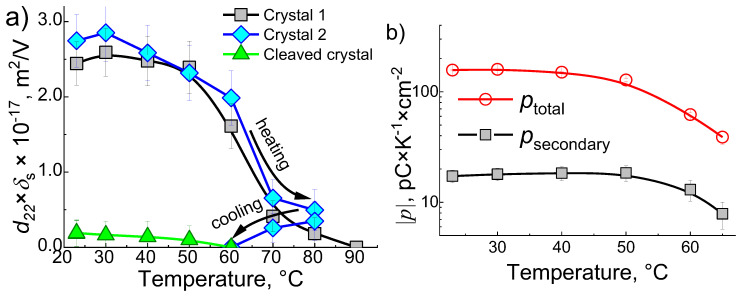
(**a**) The temperature dependence of product $d_{22}\times \delta_{s}$ for two as-grown {010} surfaces of α-gly. The graph for the cleaved crystal is shown for comparison; (**b**) The total and the secondary surface pyroelectric coefficients vs. temperature.

All as-grown {010} faces of α-gly displayed a transient, <100 msec, pyroelectric current in response to switching ON the laser illumination of the surface (Figure 1a), while the cleaved {010} faces did not generate a pyroelectric response. According the derivations given in ref. [11], the current (j) decays with time as:

(3) $$j=j_{0}\times erf\left(\sqrt{\frac{\delta_{s}^{2}}{4\cdot D\cdot t}}\right)$$

where $D=0.05{\;\mathrm{cm}}^{2}/s$ [17] is the thermal diffusion coefficient of α-gly and $j_{0}$ is the current at t = 0. Fitting the j vs. t dependence to erf-function leads to the value of $\delta_{s}$ = 288 ± 25 µm (Figure 1b), which matches the previous estimates [17]. The fact that the current-time response can be fitted with high degree of confidence to Equaion (3) at all temperatures implies (Figure 1a) that the polarization is constant within the surface layer. $\delta_{s}$ does not change noticeably with temperature upon heating to 60 °C, above which the pyroelectric response rapidly drops and at 70 °C vanishes completely, which implies that the surface layer is a well-defined structure. The temperature at which the pyroelectric response vanishes is highly reproducible with about ±4 °C variations between the samples. One has to point out that according to these measurements, the NSL has well defined thickness, constant polarization throughout and well-defined decomposition temperature, which strongly suggests that NSL is, in fact, a well-defined phase. $j_{0}$ can be expressed as:

(4) $$j_{0}=\frac{F_{d}\times p}{C_{V}\times Th_{s}}\;,$$

where $F_{d}$ is the heating power at the surface of the sample (in W) and $C_{V}$ = 1.545 J/(cm^3^×K) is the thermal capacitance per unit volume. From the value of $j_{0}$ we have deduced the total pyroelectric coefficient, p, (Figure 2b). In contrast to the thickness of the NSL, the pyroelectric coefficient decays gradually from 40 °C to zero at 70 °C. One has to point out that the pyroelectric coefficient of the NSL is fairly large for a non-ferroelectric material, ≈1.6 × $10^{-10}\;C/(m^{2}\times K)$ at 35 °C. The direction of the pyroelectric effect is always negative, which, assuming polarization decays with temperature, suggests that the polarization is directed outward of the crystal.

### 3.2. Piezoelectric Measurements

The as-grown {010} faces of α-glycine exhibit piezoelectric response, i.e., detectable surface displacement in response to applied voltage (Figure 2a). However, the cleaved {010} faces do not show any displacement, which agrees with the absence of the pyroelectric response from them. The displacement of the as-grown {010} faces in response to the applied voltage of 50–150 V was within a few pm range. The product $d_{22}\times \delta_{s}$ deduced from the displacement–temperature dependence was between 2 × $10^{-17}$ and 3 × $10^{-17}{\;m}^{2}/V$. The temperature dependence of the $d_{22}\times \delta_{s}$ closely mirrored the temperature dependence of the pyroelectric coefficient p: gradual decay starting from 40 °C and almost complete disappearance between 70 and 90 °C, indicating that there is a very narrow temperature range within which the NSL is still piezoelectric but already not pyroelectric. However, once the crystal reaches 70 °C, the decay in the piezoelectric effect becomes irreversible, even if the direction of the temperature change is reversed (Figure 2a, crystal 2). Using the value of $\delta_{s}$ deduced from the pyroelectric measurements, we have calculated the effective piezoelectric coefficient of NSL at each temperature. It is surprisingly small but still measurable, $d_{22}$, of ~0.1 pm/V. The reduction in the piezoelectric coefficient can be due to clamping of the surface layer by the rest of the crystal [18]. This effect is also responsible for the significant reduction in the piezoelectric coefficient of ferroelectric thin films as compared to their bulk counterparts [19].

It is of a particular interest to compare the total, p, and the secondary pyroelectric coefficient $p_{sec}$ = $d_{22}\times \gamma_{2}\times Y_{2}$, where $\gamma_{2}$ = 7 × $\;10^{-5}$ $K^{-1}$ is the thermal expansion coefficient of α-gly in the [010] direction [17] and $Y_{2}$ = 26 GPa is the Young’s modulus in the [010] direction [20]. While the total pyroelectric coefficient (measured at constant pressure) characterizes all contributions to the change in polarization, the secondary pyroelectric coefficient characterizes the contribution arising from the change in crystal dimensions due to thermal expansion. The difference between $p_{primary}=p-p_{sec}$ describes the changes to the overall polarization due to internal degrees of freedom, for the case of α-gly, these are the changes in state of the incorporated water molecules. The results of the calculations show that the secondary pyroelectric coefficient is only (12 ± 2)% of the total one, which is a particularly small, comparable only with that of LiTaO_3_, for which the thermal expansion coefficient in the polar direction is exceptionally small (2 × $10^{-6}$ $K^{-1}$) [21]. The relatively small secondary pyroelectric effect indicates that the polarization in the NSL is induced predominantly by the guest molecule, i.e., water, with minimum polar distortions introduced to the host lattice. As suggested earlier [1,2], the incorporation of water between the layer of α-gly may be a satisfactory explanation of the weak piezoelectricity and small secondary pyroelectric effect. This also excludes a possibility that the polarity of the NSL comes from small inclusion of β-glycine, which is, indeed, polar [22].

In addition to providing a convincing evidence on the existence of surface piezoelectric effect in the NSL of α-glycine and its nature, the data presented above suggest that stereospecific incorporation of small guest molecules (e.g., water) may take place without inducing distortions to the host lattice. Thereby, one can design a crystal with a large primary pyroelectric effect, induced by the guest, but with very low piezoelectric effect, because it originates from the host. This is very promising for the development of pyroelectric sensors in which the piezoelectric effect should be suppressed.

## 4. Conclusions

To conclude, we present a clear experimental evidence of the existence of surface piezoelectric effect in α-glycine induced by the incorporation of water molecules during the crystal growth. These data complement earlier observations of the surface pyroelectricity in glycine and provide a way for the control of piezoelectric and pyroelectric effects of crystals using stereospecific incorporation of the guest molecules.
